# Acute Ischemic Pediatric Stroke Management: An Extended Window for Mechanical Thrombectomy?

**DOI:** 10.3389/fneur.2017.00634

**Published:** 2017-11-29

**Authors:** Ashish Kulhari, Elizabeth Dorn, Jonathan Pace, Vilakshan Alambyan, Stephanie Chen, Osmond C. Wu, Macym Rizvi, Anthony Hammond, Ciro Ramos-Estebanez

**Affiliations:** ^1^Department of Neurology, Neurological Institute, University Hospitals, Cleveland, OH, United States; ^2^Department of Neurological Surgery, Neurological Institute, University Hospitals, Cleveland, OH, United States; ^3^Department of Pediatrics, Rainbow Babies and Children’s Hospital, Cleveland, OH, United States; ^4^Department of Physiology, Case Western Reserve University, Cleveland, OH, United States; ^5^Department of Emergency Medicine, University Hospitals, Cleveland, OH, United States

**Keywords:** cerebral infarction, stroke, child, restrictive cardiomyopathy, management, tissue plasminogen activator, mechanical thrombectomy

## Abstract

Ischemic stroke is a rare condition to afflict the pediatric population. Congenital cardiomyopathy represents one of several possible etiologies in children. We report a 9-year-old boy who developed right middle cerebral artery stroke secondary to primary restrictive cardiomyopathy. In the absence of pediatric guidelines, the child met adult criteria for mechanical thrombectomy given the small core infarct and large penumbra. The literature suggests children may benefit from mechanical thrombectomy in carefully selected cases. Our patient exemplifies specific circumstances in which acute stroke therapy with thrombolysis and thrombectomy may be safe.

## Background

Pediatric arterial ischemic stroke is a rare condition with an estimated incidence of 1.6/100,000 children/year (1, 2). Its diagnosis is challenging because roughly 50% of the children afflicted bear no known vascular risk factor. Low-clinical suspicion rates coupled with a high variability of presentations often lead to a significant diagnostic deferral (3). Furthermore, despite a cohort of documented risk factors, one-third of stroke diagnoses are characterized as cryptogenic (4). In essence, the absence of established treatment guidelines burdens therapeutic decision-making and possibly children’s outcomes.

Adult populations benefit from systematic acute stroke management evidence (5–8). Conversely, current pediatric guidelines are based upon weak evidence and expert consensus owing to a heterogeneous pathophysiology, and inherent enrolling limitations (9). The efficacy and safety of intravenous thrombolysis use in children is not well defined (10–12). Further therapies such as intra-arterial thrombolysis and mechanical revascularization need additional investigation. Encouraging efforts provide a framework for future prospective trials (13–16). This manuscript contributes to the current literature on successful pediatric stroke management with thrombolysis and mechanical thrombectomy and advocates for a window of opportunity for improved outcomes. Oral and written informed consent was obtained from the parents of the child whose case is herein reported.

## Introduction

A 9-year-old boy with no known past medical history presented with left hemiparesis, hemihypesthesia, and dysarthria. There had been no witnessed seizure activity. He was last noted to be at neurologic baseline 5.5 h earlier, before sleep. The initial community emergency room diagnosis was Todd’s paralysis. He received intravenous lorazepam and a loading dose of phosphenytoin. When there was no clinical improvement 2 h later, an emergent neurological consultation recommended a computed tomography (CT) of the head. The CT head demonstrated an evolving right middle cerebral artery (MCA) stroke. The presence of a “hyperdense MCA sign” on CT suggested a proximal MCA thrombus. The patient was immediately transferred to our tertiary–quaternary care center.

**Figure 1:**
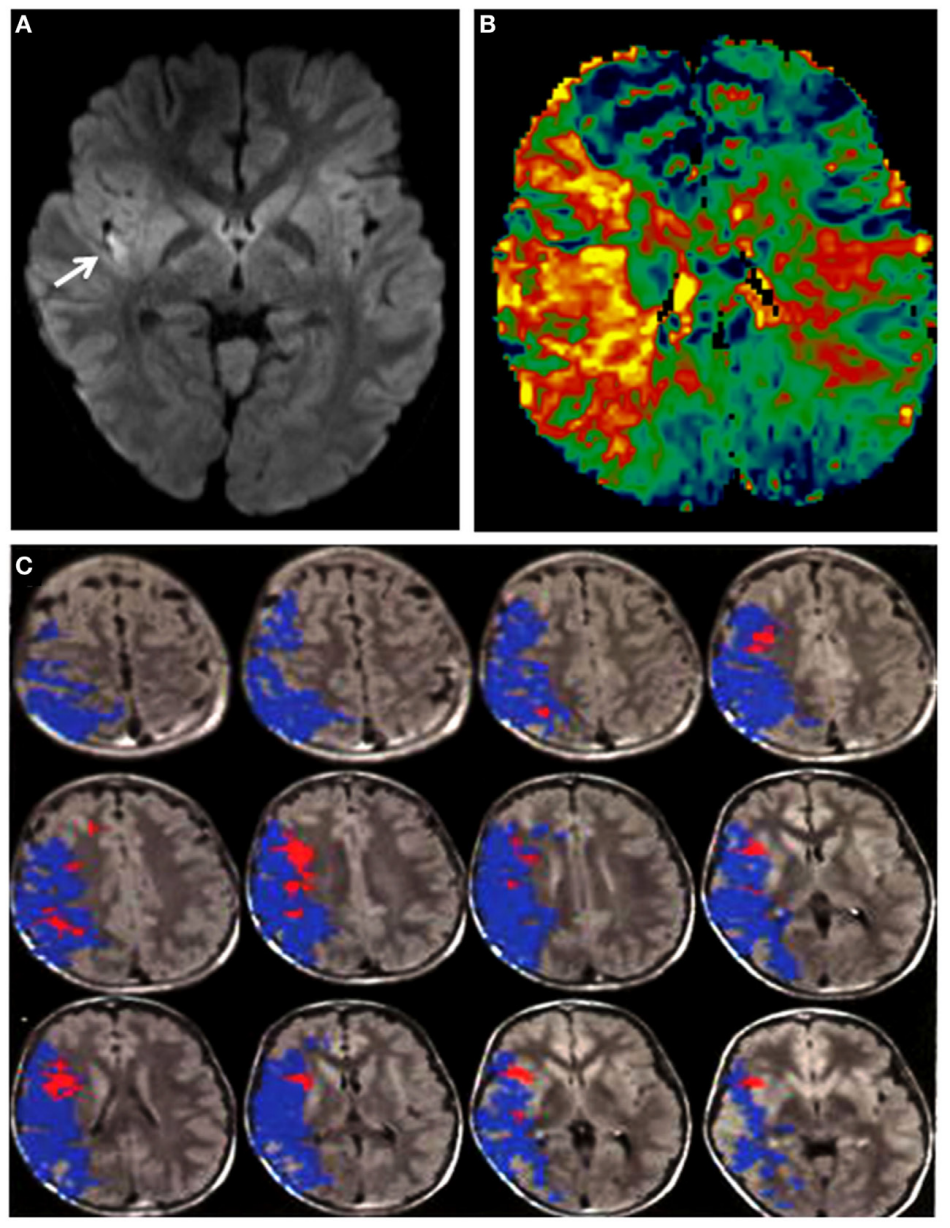
**(A)** DWI sequence showing a hyperintensity (arrow: infarct) in the right insula. **(B)** Perfusion imaging demonstrating increased mean transit time in the right middle cerebral artery (MCA) territory. **(C)** Magnetic resonance imaging with diffusion and perfusion-weighted sequences processed using Olea Sphere (Olea Medical Solutions, Inc., Cambridge, MA, USA) demonstrates volume of core infarct in red and hypoperfusion in blue, consistent with a large area of ischemic penumbra in the right MCA distribution.

On arrival, he was afebrile, with HR 120 bpm, BP 168/116, SO_2_ 99% on room air. Physical examination was notable for mild jugular venous distension and a loud P2 sound on cardiac auscultation. There was no hepatomegaly or ascites. EKG showed biatrial enlargement. He followed two-step appendicular (right-sided) commands, with an overall Pediatric National Institute of Health Stroke Scale score of 7. Brain magnetic resonance imaging (MRI) and angiogram of head and neck was significant for a right insular region infarction, as well as a right (M1 segment) MCA occlusion. Perfusion-weighted imaging was also obtained, which demonstrated significantly increased mean transit time. The stroke volume was calculated to be 6 cc by the ABC/2 method (17). A large area of perfusion deficit, coupled with small area of infarction, is what is commonly referred to as a large perfusion/diffusion mismatch (Figure 1). The large area of perfusion/diffusion mismatch observed in this patient is a relative indication for pursuing treatment.

### Management

**Figure 2:**
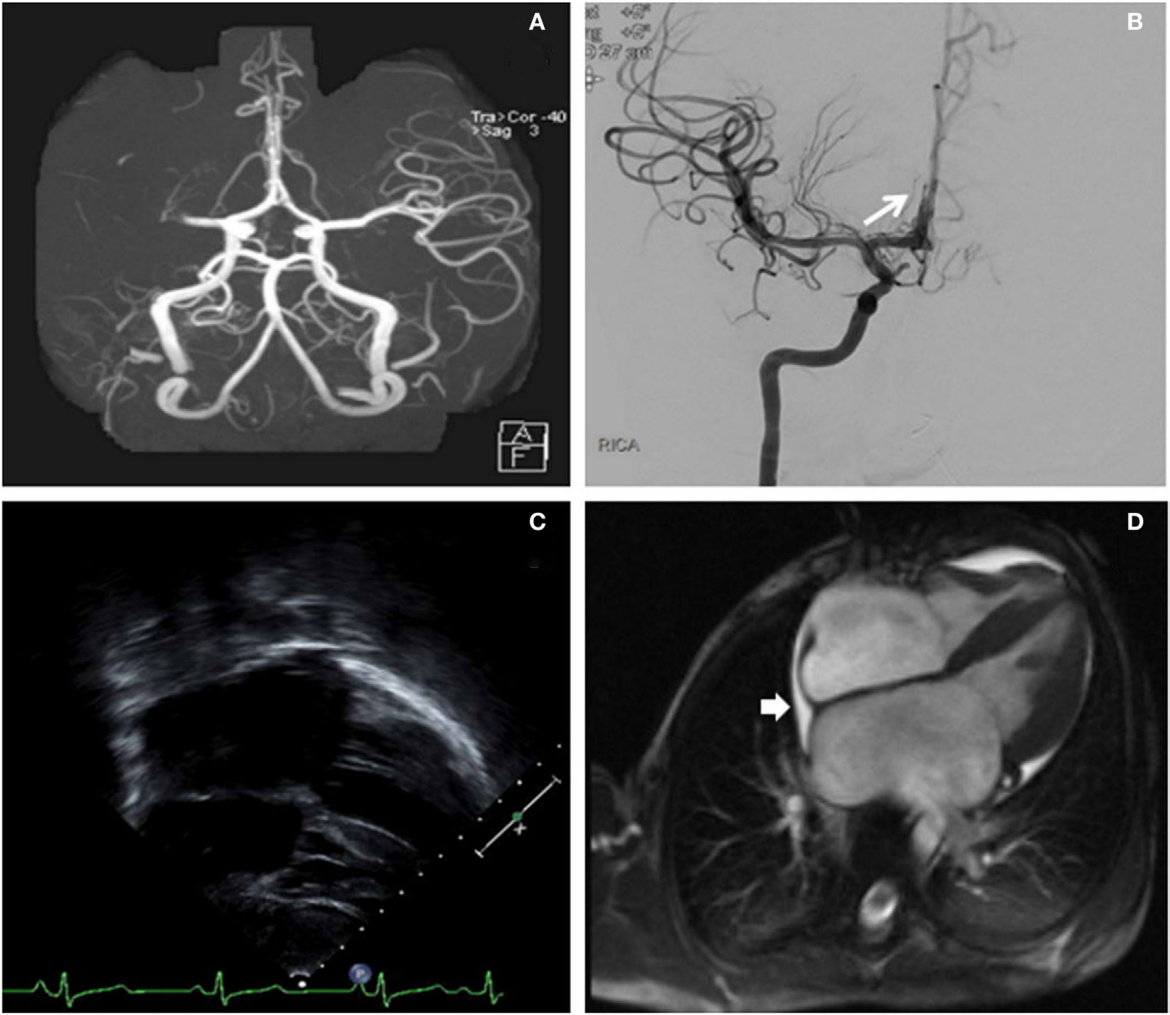
**(A)** Right middle cerebral artery (MCA) cutoff (M1) in a computed tomography angiogram (linear arrow). **(B)** Digital subtraction angiography (antero-posterior) exhibiting a recanalization of the right MCA post-thrombectomy. The long arrow identifies the thromboembolic occlusion of a distal right anterior cerebral artery segment. **(C)** Long-axis echocardiogram demonstrating biatrial enlargement. **(D)** Cardiac magnetic resonance imaging re-demonstrating the biatrial enlargement, and also revealing a small pericardial effusion (short arrow).

Seven hours after last seen at baseline, our child was significantly outside of the intravenous recombinant tissue plasminogen activator (IV r-tPA) therapeutic window. The recommended pediatric IV r-tPA window is 3–4.5 h after last known normal (9). However, he was deemed a suitable candidate for endovascular recanalization given the small core infarct and large penumbra. The patient was taken to the neuroendovascular suite and intubated. A 5 French (Fr) sheath was placed in the right common femoral artery and a 5 Fr 90 cm Envoy (Codman & Shurtleff, Inc., Raynham, MA, USA) was navigated into the distal cervical right internal carotid artery (ICA). Right ICA injections showed a right M1 segment MCA occlusion just distal to the origin of the right anterior temporal artery (Figures 2A,B). Successful mechanical thrombectomy was performed with a 3 mm × 20 mm Trevo retrievable stent device (Stryker Neurovascular, Fremont, CA, USA). Two passes with the Trevo were performed while under continuous aspiration through the base catheter during retraction of the retrievable stent. Thrombolysis in cerebral infarction (TICI) 3 right MCA recanalization was achieved within 1.5 h of completion of the MRI, for a total time between symptom onset to recanalization of 8.5 h. This procedure was complicated by thromboembolic occlusion of the A2 segment of the right anterior cerebral artery (Figure 2).

An urgent transesophageal echocardiogram confirmed bilateral severe atrial enlargement with normal ventricular size and function, with cardiac MRI confirming the diagnosis of restrictive cardiomyopathy and the absence of an intracardiac thrombus (Figure 2). A right ventricular endomyocardial biopsy showed cardiomyocyte hypertrophy with endocardial and superficial interstitial fibrosis.

Subsequently, anticoagulation with dabigatran was prescribed for secondary stroke prevention. Given his high risk for arrhythmia, an implantable cardiac defibrillator was placed. His hypercoagulable work-up was negative. Genetic testing revealed a missense mutation in the TNNI3 gene consistent with a primary restrictive cardiomyopathy (PRCM). Following the diagnosis of PRCM, a detailed history revealed frequent episodes of lip cyanosis when swimming or biking, which caused him to avoid physical exertion. This behavior had become progressively obvious to his relatives, who had attributed his inactivity to “being lazy.” On discharge, his examination was only notable for a left pronator drift (NIHSS 1), with a modified Rankin score of 1. Four months later, he underwent a successful heart transplant and at 1-year follow up, he continued to be without thromboembolic sequelae and neurologically stable.

## Discussion

### Primary Restrictive Cardiomyopathy

Primary restrictive cardiomyopathy accounts for 2.5–5% of cardiomyopathies in children. It is characterized by biatrial enlargement, normal left ventricular wall thickness and atrioventricular valves, impaired ventricular filling with restrictive physiology, and preserved systolic function (18–21). Most commonly PRCM is idiopathic in nature (20, 22). Other etiologies involve genetic disorders, skeletal myopathies, and storage diseases (23, 24).

Arteriopathy is the most common cause of pediatric stroke. Our patient presented with congenital cardiomyopathy (CCM), which may herald a neglected diagnosis, yet typically occurs in 20–30% of children undergoing corrective surgery (25, 26). Children with CCM develop a procoagulant state owing to turbulent blood flow, a malfunctioning hemostasis cascade (27–32), abnormal fibrinolysis factors (30–33), and anomalous platelet count and function (28, 30, 34, 35).

### Presentation/Diagnosis

Primary restrictive cardiomyopathy examination commonly reveals evidence of pulmonary hypertension and cor pulmonale, such as loud P2, gallop, distended jugular veins, hepatomegaly, and ascites (36–38). Abnormal EKG findings (right and/or left atrial enlargement, though ST segment depression and ST-T wave abnormalities) are present to varying extent in 98% of cases (36). Chest radiograph commonly shows cardiomegaly, particularly atrial enlargement, as well as pulmonary edema (36).

Up to 15% of children with PRCM present with arrhythmia (atrial flutter or atrial fibrillation), or conduction disorders (Mobitz 2 and third-degree AV block), or pre-excitation syndromes (e.g., Wolff–Parkinson–White syndrome) (38). Thereby, PRCM represents a known source of intracardiac thrombus formation due to blood stasis in the setting of severely dilated atrial chambers (18).

Although our patient did experience mild dyspnea on exertion, he did not exhibit other significant symptoms of cardiac decompensation, such as chest pain or syncope, and thus he remained undiagnosed until he developed symptoms secondary to his intracranial embolic stroke (36–38).

Primary restrictive cardiomyopathy is usually diagnosed by a combination of echocardiogram and cardiac catheterization, with the latter being the definitive test (18, 39–42). Elevation of biventricular end diastolic pressures in addition to pulmonary hypertension on cardiac catheterization is suggestive of PRCM (18, 39–42). Endomyocardial biopsy was non-diagnostic (18, 22, 43).

### State of the Art

Adult acute arterial ischemic stroke management guidelines unequivocally support the use of IV r-tPA (44, 45), endovascular mechanical thrombectomy, or intra-arterial r-tPA administration (46, 47) in specific situations.

The Thrombolysis in Pediatric Stroke (TIPS) trial (9) and latest Royal College of Pediatrics and Child Health (RCPCH) recommendations (48) represent the current landmark in acute pediatric stroke. Pediatricians remain cautiously optimistic in the light of adult thrombolysis therapeutic success. Despite substantial differences in thrombolytic pharmacokinetics and dose–response (49, 50), IV r-tPA in children can be deemed safe through a focus on meticulous selection criteria (9, 51). A majority of childhood strokes are due to intracranial arteriopathy, wherein mechanical thrombectomy is questionable, owing to the inflamed and friable arterial walls (52). Conversely, cardio-embolic strokes represent an ideal correlate to adults that would benefit from mechanical intervention. However, the inability to use thrombo-aspiration devices due to small artery caliber and the consequent risk of thrombus fragmentation or migration may detract from enthusiasm.

While recent data have begun to unravel the detrimental effect of aging on collateral supply (53–55), pediatric collateral flow remains largely unexplored. Sparse reports compare pediatric stroke outcomes of similar NIHSS severity yet distinct collateral profiles treated with endovascular therapy (56). Nevertheless, the latest adult data support superior TICI scores (57–59) and functional outcomes (60–64) in patients undergoing recanalization in the setting of better collateral flow. Moreover, recanalization seems to carry fewer rates of hemorrhagic conversion post-instrumentation (57, 65, 66). At this time, the relationship of efficient pediatric cortical collaterals with diffusion/perfusion mismatch is incompletely understood. Unfavorable imaging does not render infarct progression an absolute certainty without treatment (67). Therefore, it would be intuitive to measure the recanalization benefit in clinical terms until radiological (i.e., angiographic) or parenchymal metrics are validated in children (68). In a field without studies controlled for intervention in the presence of good collaterals (69, 70), the preceding evidence informed the management of our patient.

With the aforementioned limitations to translate adult evidence to children in mind, we believe our case and others (71–77) provide hope and opportunity. Our child presented outside of the 4.5-h IV r-tPA window adopted by the TIPS trial methodology which represents the standard of practice in both children and adults. Additionally, the onset-puncture time was beyond our institutional guideline of 8 h yet within the current maximum advisory of 12 h by RCPCH. Nevertheless, the patient had a rich collateral supply, allowing for a small core infarct and large penumbra to be seen upon imaging, which led to our decision to intervene (Figure 1). Therefore, we pursued mechanical thrombectomy and salvaged a large ischemic brain area with a great impact on the child’s functional status. In terms of secondary prevention, we prescribed dabigatran which is a direct thrombin inhibitor that is independent of the variable antithrombin levels encountered in pediatrics and offers a favorable pharmacological profile (78). Primarily to avoid age-appropriate inconsistencies in daily intake, interactions with other medications, potential osteoporosis in long-term use of warfarin (79), and frequent laboratory monitoring (10, 80–82). Finally, our child underwent heart transplantation (18, 22, 39, 41, 42, 83, 84), which will prevent the occurrence of cardioembolic events.

## Conclusion

Pediatric ischemic stroke is an under-recognized condition. We highlight stroke as a potentially devastating and treatable condition in children presenting with acute neurologic deficits. Owing to the possibility of sufficient collateral circulations and smaller core infarct volumes, children might benefit from an extended therapeutic window for mechanical thrombectomy beyond 5 h.

## Informed Consent

A written informed consent was obtained from the parents for the publication of this report.

## Author Contributions

AK conceptualized, drafted, and critically revised the manuscript. ED analyzed the data, critiqued, and revised the manuscript. JP acquired the data, critically reviewed, and revised the manuscript. VA reviewed the literature, analyzed the data, and critically revised the manuscript. SC reviewed the literature and critically revised the manuscript. OW, MR, and AH analyzed the data and critically revised the manuscript. CR-E conceptualized and outlined the manuscript, oversaw data acquisition, supervised the initial drafting, and critically reviewed the manuscript. All authors approved the final manuscript as submitted and agreed to be accountable for all aspects of the work.

## Conflict of Interest Statement

The authors declare that the research was conducted in the absence of any commercial or financial relationships that could be construed as a potential conflict of interest.

## Abbreviations

CCM, congenital cardiomyopathy; CHD, congenital heart disease; CT, computed tomography; ECMO, extracorporeal membrane oxygenation; MCA, middle cerebral artery; MRA, magnetic resonance angiography; MRI, magnetic resonance imaging; NIHSS, National Institute of Health Stroke Scale; PRCM, primary restrictive cardiomyopathy; TICI, thrombolysis in cerebral infarction; TIPS, Thrombolysis in Pediatric Stroke; r-tPA, recombinant tissue plasminogen activator; IV, intravenous; RCPCH, Royal College of Pediatrics and Child Health.

## References

[1] Mittal SO ThatiGanganna S Kuhns B Strbian D Sundararajan S Acute ischemic stroke in pediatric patients. Stroke (2015) 46(2):e32–4. 10.1161/STROKEAHA.114.007681 25503551

[2] Mallick AA Ganesan V Kirkham FJ Fallon P Hedderly T McShane T Childhood arterial ischaemic stroke incidence, presenting features, and risk factors: a prospective population-based study. Lancet Neurol (2014) 13(1):35–43. 10.1016/S1474-4422(13)70290-4 24304598

[3] Mallick AA Ganesan V Kirkham FJ Fallon P Hedderly T McShane T Diagnostic delays in paediatric stroke. J Neurol Neurosurg Psychiatry (2015) 86(8):917–21. 10.1136/jnnp-2014-309188 25342203

[4] Per H Unal E Poyrazoglu HG Ozdemir MA Donmez H Gumus H Childhood stroke: results of 130 children from a reference center in Central Anatolia, Turkey. Pediatr Neurol (2014) 50(6):595–600. 10.1016/j.pediatrneurol.2013.12.023 24842257

[5] Lansberg MG O’Donnell MJ Khatri P Lang ES Nguyen-Huynh MN Schwartz NE Antithrombotic and thrombolytic therapy for ischemic stroke: antithrombotic therapy and prevention of thrombosis, 9th ed: American College of Chest Physicians Evidence-Based Clinical Practice Guidelines. Chest (2012) 141(2 Suppl):e601S–e36S. 10.1378/chest.11-2302 22315273 PMC3278065

[6] Jauch EC Saver JL Adams HP Bruno A Connors JJ Demaerschalk BM Guidelines for the early management of patients with acute ischemic stroke. A guideline for healthcare professionals from the American Heart Association/American Stroke Association. Stroke (2013) 44(3):870–947. 10.1161/STR.0b013e318284056a 23370205

[7] Powers WJ Derdeyn CP Biller J Coffey CS Hoh BL Jauch EC 2015 American Heart Association/American Stroke Association Focused update of the 2013 guidelines for the early management of patients with acute ischemic stroke regarding endovascular treatment: a guideline for healthcare professionals from the American Heart Association/American Stroke Association. Stroke (2015) 46(10):3020–35. 10.1161/STR.0000000000000074 26123479

[8] Furlan AJ Endovascular therapy for stroke – it’s about time. N Engl J Med (2015) 372(24):2347–9. 10.1056/NEJMe1503217 25882509

[9] Rivkin MJ deVeber G Ichord RN Kirton A Chan AK Hovinga CA Thrombolysis in Pediatric Stroke Study. Stroke (2015) 46(3):880–5. 10.1161/strokeaha.114.008210 25613306 PMC4342311

[10] Monagle P Chan AK Goldenberg NA Ichord RN Journeycake JM Nowak-Gottl U Antithrombotic therapy in neonates and children: antithrombotic therapy and prevention of thrombosis, 9th ed: American College of Chest Physicians Evidence-Based Clinical Practice Guidelines. Chest (2012) 141(2 Suppl):737S–801S. 10.1378/chest.11-2308 22315277 PMC3278066

[11] Rivkin MJ Bernard TJ Dowling MM Amlie-Lefond C. Guidelines for urgent management of stroke in children. Pediatr Neurol (2016) 56:8–17. 10.1016/j.pediatrneurol.2016.01.016 26969237

[12] Roach ES Golomb MR Adams R Biller J Daniels S deVeber G Management of stroke in infants and children. A scientific statement from a special writing group of the American Heart Association Stroke Council and the Council on Cardiovascular Disease in the Young. Stroke (2008) 39(9):2644–91. 10.1161/strokeaha.108.189696 18635845

[13] Wilson JL Eriksson CO Williams CN. Endovascular therapy in pediatric stroke: utilization, patient characteristics, and outcomes. Pediatr Neurol (2017) 69:87–92.e2. 10.1016/j.pediatrneurol.2017.01.013 28233666 PMC6394403

[14] Amlie-Lefond C deVeber G Chan AK Benedict S Bernard T Carpenter J Use of alteplase in childhood arterial ischaemic stroke: a multicentre, observational, cohort study. Lancet Neurol (2009) 8(6):530–6. 10.1016/S1474-4422(09)70106-1 19423401

[15] Lehman LL Kleindorfer DO Khoury JC Alwell K Moomaw CJ Kissela BM Potential eligibility for recombinant tissue plasminogen activator therapy in children: a population-based study. J Child Neurol (2011) 26(9):1121–5. 10.1177/0883073811408091 21628693 PMC3420804

[16] Demaerschalk BM Kleindorfer DO Adeoye OM Demchuk AM Fugate JE Grotta JC Scientific rationale for the inclusion and exclusion criteria for intravenous alteplase in acute ischemic stroke: a statement for healthcare professionals from the American Heart Association/American Stroke Association. Stroke (2016) 47(2):581–641. 10.1161/STR.0000000000000086 26696642

[17] Sims JR Gharai LR Schaefer PW Vangel M Rosenthal ES Lev MH ABC/2 for rapid clinical estimate of infarct, perfusion, and mismatch volumes. Neurology (2009) 72(24):2104–10. 10.1212/WNL.0b013e3181aa5329 19528517 PMC2697964

[18] Denfield SW Rosenthal G Gajarski RJ Bricker JT Schowengerdt KO Price JK Restrictive cardiomyopathies in childhood. Etiologies and natural history. Tex Heart Inst J (1997) 24(1):38–44. 9068138 PMC325396

[19] Lewis AB. Clinical profile and outcome of restrictive cardiomyopathy in children. Am Heart J (1992) 123(6):1589–93. 10.1016/0002-8703(92)90814-C 1595540

[20] Maron BJ Towbin JA Thiene G Antzelevitch C Corrado D Arnett D Contemporary definitions and classification of the cardiomyopathies: an American Heart Association Scientific Statement from the Council on Clinical Cardiology, Heart Failure and Transplantation Committee; Quality of Care and Outcomes Research and Functional Genomics and Translational Biology Interdisciplinary Working Groups; and Council on Epidemiology and Prevention. Circulation (2006) 113(14):1807–16. 10.1161/CIRCULATIONAHA.106.174287 16567565

[21] Nugent AW Daubeney PE Chondros P Carlin JB Cheung M Wilkinson LC The epidemiology of childhood cardiomyopathy in Australia. N Engl J Med (2003) 348(17):1639–46. 10.1056/NEJMoa021737 12711738

[22] Denfield SW Webber SA. Restrictive cardiomyopathy in childhood. Heart Fail Clin (2010) 6(4):445–52, viii. 10.1016/j.hfc.2010.05.005 20869645

[23] Kaski JP Syrris P Burch M Tome-Esteban MT Fenton M Christiansen M Idiopathic restrictive cardiomyopathy in children is caused by mutations in cardiac sarcomere protein genes. Heart (2008) 94(11):1478–84. 10.1136/hrt.2007.134684 18467357

[24] Ware SM Quinn ME Ballard ET Miller E Uzark K Spicer RL. Pediatric restrictive cardiomyopathy associated with a mutation in beta-myosin heavy chain. Clin Genet (2008) 73(2):165–70. 10.1111/j.1399-0004.2007.00939.x 18076673

[25] Mahle WT Tavani F Zimmerman RA Nicolson SC Galli KK Gaynor JW An MRI study of neurological injury before and after congenital heart surgery. Circulation (2002) 106(12 Suppl 1):I109–14. 10.1161/01.cir.0000032908.33237.b1 12354718

[26] McQuillen PS Barkovich AJ Hamrick SE Perez M Ward P Glidden DV Temporal and anatomic risk profile of brain injury with neonatal repair of congenital heart defects. Stroke (2007) 38(2 Suppl):736–41. 10.1161/01.STR.0000247941.41234.90 17261728

[27] Dennis LH Stewart JL Conrad ME A consumption coagulation defect in congenital cyanotic heart disease and its treatment with heparin. J Pediatr (1967) 71(3):407–10. 10.1016/S0022-3476(67)80302-0 6034791

[28] Horigome H Hiramatsu Y Shigeta O Nagasawa T Matsui A. Overproduction of platelet microparticles in cyanotic congenital heart disease with polycythemia. J Am Coll Cardiol (2002) 39(6):1072–7. 10.1016/S0735-1097(02)01718-7 11897453

[29] Ihenacho HN Fletcher DJ Breeze GR Stuart J Consumption coagulopathy in congenital heart-disease. Lancet (1973) 1(7797):231–4. 10.1016/S0140-6736(73)90069-X 4119378

[30] Jahangiri M Kreutzer J Zurakowski D Bacha E Jonas RA. Evaluation of hemostatic and coagulation factor abnormalities in patients undergoing the Fontan operation. J Thorac Cardiovasc Surg (2000) 120(4):778–82. 10.1067/mtc.2000.108903 11003762

[31] Komp DM Sparrow AW Polycythemia in cyanotic heart disease – a study of altered coagulation. J Pediatr (1970) 76(2):231–6. 10.1016/S0022-3476(70)80167-6 5410171

[32] Levin E Wu J Devine DV Alexander J Reichart C Sett S Hemostatic parameters and platelet activation marker expression in cyanotic and acyanotic pediatric patients undergoing cardiac surgery in the presence of tranexamic acid. Thromb Haemost (2000) 83(1):54–9. 10669155

[33] Goel M Shome DK Singh ZN Bhattacharjee J Khalil A. Haemostatic changes in children with cyanotic and acyanotic congenital heart disease. Indian Heart J (2000) 52(5):559–63. 11256779

[34] Colon-Otero G Gilchrist GS Holcomb GR Ilstrup DM Bowie EJ. Preoperative evaluation of hemostasis in patients with congenital heart disease. Mayo Clin Proc (1987) 62(5):379–85. 10.1016/S0025-6196(12)65442-1 3573826

[35] Maurer HM McCue CM Robertson LW Haggins JC. Correction of platelet dysfunction and bleeding in cyanotic congenital heart disease by simple red cell volume reduction. Am J Cardiol (1975) 35(6):831–5. 10.1016/0002-9149(75)90119-8 48335

[36] Denfield SW Sudden death in children with restrictive cardiomyopathy. Card Electrophysiol Rev (2002) 6(1–2):163–7. 10.1023/A:1017980331651 11984040

[37] Fitzpatrick AP Shapiro LM Rickards AF Poole-Wilson PA. Familial restrictive cardiomyopathy with atrioventricular block and skeletal myopathy. Br Heart J (1990) 63(2):114–8. 10.1136/hrt.63.2.114 2317404 PMC1024337

[38] Rivenes SM Kearney DL Smith EO Towbin JA Denfield SW. Sudden death and cardiovascular collapse in children with restrictive cardiomyopathy. Circulation (2000) 102(8):876–82. 10.1161/01.CIR.102.8.876 10952956

[39] Cetta F O’Leary PW Seward JB Driscoll DJ. Idiopathic restrictive cardiomyopathy in childhood: diagnostic features and clinical course. Mayo Clin Proc (1995) 70(7):634–40. 10.4065/70.7.634 7791385

[40] Hughes ML Kleinert S Keogh A Macdonald P Wilkinson JL Weintraub RG. Pulmonary vascular resistance and reactivity in children with end-stage cardiomyopathy. J Heart Lung Transplant (2000) 19(7):701–4. 10.1016/S1053-2498(00)00118-2 10930820

[41] Kimberling MT Balzer DT Hirsch R Mendeloff E Huddleston CB Canter CE. Cardiac transplantation for pediatric restrictive cardiomyopathy: presentation, evaluation, and short-term outcome. J Heart Lung Transplant (2002) 21(4):455–9. 10.1016/S1053-2498(01)00400-4 11927222

[42] Weller RJ Weintraub R Addonizio LJ Chrisant MR Gersony WM Hsu DT. Outcome of idiopathic restrictive cardiomyopathy in children. Am J Cardiol (2002) 90(5):501–6. 10.1016/S0002-9149(02)02522-5 12208410

[43] Cooper LT Baughman KL Feldman AM Frustaci A Jessup M Kuhl U The role of endomyocardial biopsy in the management of cardiovascular disease: a scientific statement from the American Heart Association, the American College of Cardiology, and the European Society of Cardiology. Endorsed by the Heart Failure Society of America and the Heart Failure Association of the European Society of Cardiology. J Am Coll Cardiol (2007) 50(19):1914–31. 10.1016/j.jacc.2007.09.008 17980265

[44] Brott T Broderick J Kothari R O’Donoghue M Barsan W Tomsick T Tissue plasminogen activator for acute ischemic stroke. The National Institute of Neurological Disorders and Stroke rt-PA Stroke Study Group. N Engl J Med (1995) 333(24):1581–7. 10.1056/NEJM199512143332401 7477192

[45] Furlan A Higashida R Wechsler L Gent M Rowley H Kase C Intra-arterial prourokinase for acute ischemic stroke. The PROACT II study: a randomized controlled trial. Prolyse in Acute Cerebral Thromboembolism. JAMA (1999) 282(21):2003–11. 10.1001/jama.282.21.2003 10591382

[46] Goyal M Demchuk AM Menon BK Eesa M Rempel JL Thornton J Randomized assessment of rapid endovascular treatment of ischemic stroke. N Engl J Med (2015) 372(11):1019–30. 10.1056/NEJMoa1414905 25671798

[47] Jovin TG Chamorro A Cobo E de Miquel MA Molina CA Rovira A Thrombectomy within 8 hours after symptom onset in ischemic stroke. N Engl J Med (2015) 372(24):2296–306. 10.1056/NEJMoa1503780 25882510

[48] Stroke in Childhood. Clinical Guidelines for Diagnosis, Management and Rehabilitation. (2017). Available from: http://www.rcpch.ac.uk/stroke-guideline

[49] Parmar N Albisetti M Berry LR Chan AK. The fibrinolytic system in newborns and children. Clin Lab (2006) 52(3–4):115–24. 16584057

[50] Guzzetta NA Miller BE. Principles of hemostasis in children: models and maturation. Paediatr Anaesth (2011) 21(1):3–9. 10.1111/j.1460-9592.2010.03410.x 20849450

[51] Hu YC Chugh C Jeevan D Gillick JL Marks S Stiefel MF. Modern endovascular treatments of occlusive pediatric acute ischemic strokes: case series and review of the literature. Childs Nerv Syst (2014) 30(5):937–43. 10.1007/s00381-013-2313-3 24212331

[52] Moharir M Deveber G Pediatric arterial ischemic stroke. Continuum (2014) 20(2 Cerebrovascular Disease):370–86. 10.1212/01.CON.0000446107.74796.a0 24699487 PMC10564069

[53] Malik N Hou Q Vagal A Patrie J Xin W Michel P Demographic and clinical predictors of leptomeningeal collaterals in stroke patients. J Stroke Cerebrovasc Dis (2014) 23(8):2018–22. 10.1016/j.jstrokecerebrovasdis.2014.02.018 25088172

[54] Arsava EM Vural A Akpinar E Gocmen R Akcalar S Oguz KK The detrimental effect of aging on leptomeningeal collaterals in ischemic stroke. J Stroke Cerebrovasc Dis (2014) 23(3):421–6. 10.1016/j.jstrokecerebrovasdis.2013.03.014 23583014

[55] Menon BK Smith EE Coutts SB Welsh DG Faber JE Goyal M Leptomeningeal collaterals are associated with modifiable metabolic risk factors. Ann Neurol (2013) 74(2):241–8. 10.1002/ana.23906 23536377 PMC3836863

[56] Arnold M Steinlin M Baumann A Nedeltchev K Remonda L Moser SJ Thrombolysis in childhood stroke: report of 2 cases and review of the literature. Stroke (2009) 40(3):801–7. 10.1161/STROKEAHA.108.529560 19118242

[57] Bang OY Saver JL Kim SJ Kim GM Chung CS Ovbiagele B Collateral flow predicts response to endovascular therapy for acute ischemic stroke. Stroke (2011) 42(3):693–9. 10.1161/STROKEAHA.110.595256 21233472 PMC3051344

[58] Marks MP Lansberg MG Mlynash M Olivot JM Straka M Kemp S Effect of collateral blood flow on patients undergoing endovascular therapy for acute ischemic stroke. Stroke (2014) 45(4):1035–9. 10.1161/strokeaha.113.004085 24569816 PMC4396867

[59] Nambiar V Sohn SI Almekhlafi MA Chang HW Mishra S Qazi E CTA collateral status and response to recanalization in patients with acute ischemic stroke. AJNR Am J Neuroradiol (2014) 35(5):884–90. 10.3174/ajnr.A3817 24371030 PMC7964545

[60] Miteff F Levi CR Bateman GA Spratt N McElduff P Parsons MW. The independent predictive utility of computed tomography angiographic collateral status in acute ischaemic stroke. Brain (2009) 132(8):2231–8. 10.1093/brain/awp155 19509116

[61] Lansberg MG Straka M Kemp S Mlynash M Wechsler LR Jovin TG Magnetic resonance imaging profile and response to endovascular reperfusion: results of the DEFUSE 2 Prospective Cohort Study. Lancet Neurol (2012) 11(10):860–7. 10.1016/s1474-4422(12)70203-x 22954705 PMC4074206

[62] Liebeskind DS Jahan R Nogueira RG Zaidat OO Saver JL SWIFT Investigators. Impact of collaterals on successful revascularization in solitaire FR with the intention for thrombectomy. Stroke (2014) 45(7):2036–40. 10.1161/strokeaha.114.004781 24876081 PMC4157911

[63] Lima FO Furie KL Silva GS Lev MH Camargo ÉCS Singhal AB The pattern of leptomeningeal collaterals on CT angiography is a strong predictor of long-term functional outcome in stroke patients with large vessel intracranial occlusion. Stroke (2010) 41(10):2316–22. 10.1161/strokeaha.110.592303 20829514 PMC4939434

[64] Tan IYL Demchuk AM Hopyan J Zhang L Gladstone D Wong K CT angiography clot burden score and collateral score: correlation with clinical and radiologic outcomes in acute middle cerebral artery infarct. AJNR Am J Neuroradiol (2009) 30(3):525–31. 10.3174/ajnr.A1408 19147716 PMC7051470

[65] Campbell BCV Christensen S Butcher KS Gordon I Parsons MW Desmond PM Regional very low cerebral blood volume predicts hemorrhagic transformation better than diffusion-weighted imaging volume and thresholded apparent diffusion coefficient in acute ischemic stroke. Stroke (2010) 41(1):82–8. 10.1161/strokeaha.109.562116 19959537

[66] Kidwell CS Saver JL Mattiello J Starkman S Vinuela F Duckwiler G Diffusion-perfusion MRI characterization of post-recanalization hyperperfusion in humans. Neurology (2001) 57(11):2015–21. 10.1212/WNL.57.11.2015 11739819

[67] Boardman JP Ganesan V Rutherford MA Saunders DE Mercuri E Cowan F. Magnetic resonance image correlates of hemiparesis after neonatal and childhood middle cerebral artery stroke. Pediatrics (2005) 115(2):321–6. 10.1542/peds.2004-0427 15687439

[68] Tomsick TTIMI TIBI TICI I came, I saw, I got confused. AJNR Am J Neuroradiol (2007) 28(2):382–4. 17297017 PMC7977395

[69] Goyal M Menon BK Coutts SB Hill MD Demchuk AM. Effect of baseline CT scan appearance and time to recanalization on clinical outcomes in endovascular thrombectomy of acute ischemic strokes. Stroke (2011) 42(1):93–7. 10.1161/STROKEAHA.110.594481 21088240

[70] Sheth SA Liebeskind DS. Collaterals in endovascular therapy for stroke. Curr Opin Neurol (2015) 28(1):10–5. 10.1097/WCO.0000000000000166 25514251

[71] Tsivgoulis G Horton JA Ness JM Patterson D Brethour M Abanses JC Intravenous thrombolysis followed by intra-arterial thrombolysis and mechanical thrombectomy for the treatment of pediatric ischemic stroke. J Neurol Sci (2008) 275(1–2):151–3. 10.1016/j.jns.2008.07.018 18722630

[72] Higashi K Honda T Tateno S Kawasoe Y Niwa K Matsuda S Successful selective intra-arterial thrombolytic therapy for embolic stroke in a patient with asplenia syndrome and unrepaired cyanotic congenital heart disease. Congenit Heart Dis (2006) 1(6):343–5. 10.1111/j.1747-0803.2006.00061.x 18377507

[73] Byrnes JW Williams B Prodhan P Erdem E James C Williamson R Successful intra-arterial thrombolytic therapy for a right middle cerebral artery stroke in a 2-year-old supported by a ventricular assist device. Transpl Int (2012) 25(3):e31–3. 10.1111/j.1432-2277.2011.01411.x 22211986

[74] Tan M Armstrong D Birken C Bitnun A Caldarone CA Cox P Bacterial endocarditis in a child presenting with acute arterial ischemic stroke: should thrombolytic therapy be absolutely contraindicated? Dev Med Child Neurol (2009) 51(2):151–4. 10.1111/j.1469-8749.2008.03188.x 19191846

[75] Rosman NP Adhami S Mannheim GB Katz NP Klucznik RP Muriello MA Basilar artery occlusion in children: misleading presentations, “locked-in” state, and diagnostic importance of accompanying vertebral artery occlusion. J Child Neurol (2003) 18(7):450–62. 10.1177/08830738030180070601 12940650

[76] Bhatt A Naravetla B Farooq MU Majid A Kassab M Gupta R. Treatment of a basilar artery occlusion with intra-arterial thrombolysis in a 3-year-old girl. Neurocrit Care (2008) 9(3):357–60. 10.1007/s12028-008-9086-7 18389181

[77] Benedict SL Ni OK Schloesser P White KS Bale JF Jr. Intra-arterial thrombolysis in a 2-year-old with cardioembolic stroke. J Child Neurol (2007) 22(2):225–7. 10.1177/0883073807300296 17621489

[78] Dabbous MK Sakr FR Malaeb DN Anticoagulant therapy in pediatrics. J Basic Clin Pharm (2014) 5(2):27–33. 10.4103/0976-0105.134947 25031496 PMC4074692

[79] Barnes C Newall F Ignjatovic V Wong P Cameron F Jones G Reduced bone density in children on long-term warfarin. Pediatr Res (2005) 57(4):578–81. 10.1203/01.PDR.0000155943.07244.04 15695604

[80] Andrew M Marzinotto V Brooker LA Adams M Ginsberg J Freedom R Oral anticoagulation therapy in pediatric patients: a prospective study. Thromb Haemost (1994) 71(3):265–9. 8029786

[81] Christensen TD Attermann J Hjortdal VE Maegaard M Hasenkam JM. Self-management of oral anticoagulation in children with congenital heart disease. Cardiol Young (2001) 11(3):269–76. 10.1017/S1047951101000282 11388620

[82] Marzinotto V Monagle P Chan A Adams M Massicotte P Leaker M Capillary whole blood monitoring of oral anticoagulants in children in outpatient clinics and the home setting. Pediatr Cardiol (2000) 21(4):347–52. 10.1007/s002460010078 10865011

[83] Chen SC Balfour IC Jureidini S. Clinical spectrum of restrictive cardiomyopathy in children. J Heart Lung Transplant (2001) 20(1):90–2. 10.1016/S1053-2498(00)00162-5 11166616

[84] Fenton MJ Chubb H McMahon AM Rees P Elliott MJ Burch M Heart and heart-lung transplantation for idiopathic restrictive cardiomyopathy in children. Heart (2006) 92(1):85–9. 10.1136/hrt.2004.049502 16365357 PMC1860993

